# Langerhans cell histiocytosis on the penis: a case report

**DOI:** 10.1186/1471-2490-6-28

**Published:** 2006-10-11

**Authors:** Jun Hagiuda, Munehisa Ueno, Satoshi Ashimine, Isao Kuroda, Keisuke Ishizawa, Nobuhiro Deguchi

**Affiliations:** 1 Department of Urology, Saitama Medical University, Saitama, Japan; 2 Department of Pathology, Saitama Medical University, Saitama, Japan

## Abstract

**Background:**

Langerhans cell histiocytosis affects mainly young children and features an accumulation of CD1a+ dendritic Langerhans cells in the bone, skin, and other organs. A few cases of Langerhans cell histiocytosis on the penis have been reported in the literature. We present a case of Langerhans cell histiocytosis on the penis and review the similar cases in the literature.

**Case presentation:**

The patient was a 13-year-old boy who had a history of lymph node, femur bone, and pituitary-thalmic axis lesions from Langerhans cell histiocytosis who noticed a painful nodule on the prepuce of his penis. The histological and immunohistochemical examination fulfilled the criteria of Langerhans cell histiocytosis.

**Conclusion:**

We herein describe the case reported of Langerhans cell histiocytosis on the penis.

## Background

Langerhans cell histiocytosis (LCH) is characterized by an organ-specific infiltration of cells with many morphological features and immunohistochemical markers of Langerhans cells. Clinically, LCH ranges from self-healing lesions to a multi-system involvement with organ dysfunction resistant to current therapies. The lesions appear in multiple organs, for example in the bones, skin, and lungs, but a lesion localized on the penis is uncommon. We herein report a case of LCH on the penis.

## Case presentation

**Figure 1:**
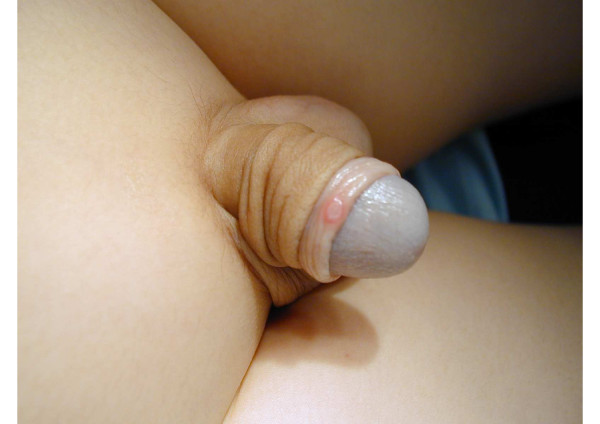
Painful nodule on the prepuce of the penis.

**Figure 2:**
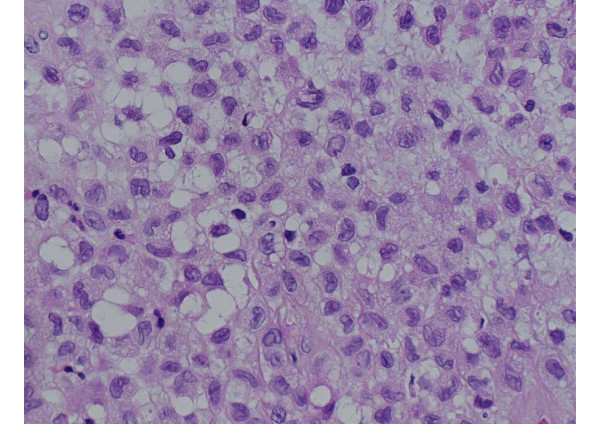
High-power view showing mononuclear Langerhans cells. The irregular nuclei have prominent grooves and folds. Numerous eosinophils can also be seen. Hematoxylin and eosin staining ×400.

**Figure 3:**
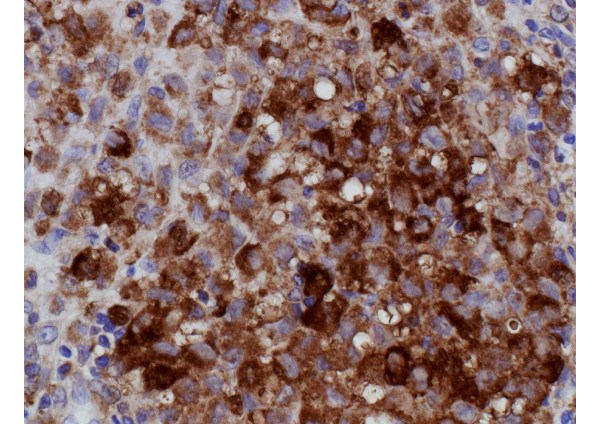
Note membranous immunoreactivity of S-100 protein and CD1a in the majority of the Langerhans cells. ×400.

**Figure 4:**
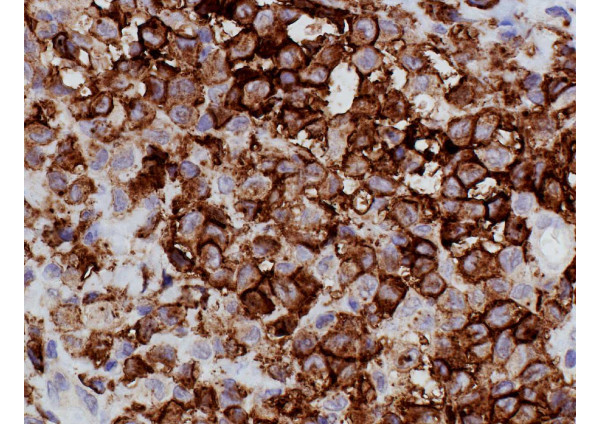
Note membranous immunoreactivity of S-100 protein and CD1a in the majority of the Langerhans cells. ×400

A 13-year-old boy presented with a nodular lesion on the prepuce of his penis. A diagnosis of LCH had been made at the age of 3 years. The first lesion was on a cervical lymph node. After the administration of chemotherapy with vincristine, cyclophosphamide, and predonisone, the lesion resolved. When the patient was 11 years old, he restarted chemotherapy (cyclophosphamide and 6-mercaptopurine) because of a recurrent lesion in the femur bone and along the pituitary-thalmic axis. At this time, he noticed a painful nodule, 5 mm in diameter, which was elastic, hard, and reddish, with a smooth surface (Fig. 1). Surgical resection of the nodule in the foreskin, without amputation or circumcision, was performed. Histologically, the lesions consisted of diffuse infiltrates of Langerhans cells with indented or grooved nuclei and eosinophilic or pale cytoplasm. Also present were various numbers of eosinophils, lymphocytes, and neutrophils. The lesions represented stromal edema and mild fibrosis. Their surface was focally eroded. Scattered vascular proliferation was present (Fig. 2). Both S-100 protein and CD1a were immunoreactive in the majority of these Langerhans cells (Fig. 3, 4). These findings led to a diagnosis of LCH of the penis. An additional treatment with oral and ointment steroid was given, and the patient was well throughout a 4-month follow-up, showing no signs of other lesions.

## Conclusion

Histiocytosis X, which included Letterer-Siwe's disease, Hand-Shuller-Christian's disease, and eosinophilic granuloma, was renamed Langerhans cell histiocytosis in 1985 by the Histiocytic Society [1]. The diagnosis of LCH has been based on a histopathological pattern in biopsy specimens showing mono- or multinucleated Langerhans cells, histiocytes, and eosinophils. The presence of Birbeck granules on electron microscopic examination or of antigenic markers that react with CD1a glycoprotein and the cytoplasmic protein S100 detected by immunostain is considered diagnostic, as shown in the present case. LCH lesions are common in the bone, lung, skin, liver, spleen, and lymph nodes. In 30.6% of patients, LCH involved more than one body system. Twenty-five percent of patients had skin and/or mucous membrane LCH. The most common mucous membranes involved are the genitalia and oral mucous [2]. In the current literature, we found only 6 cases of a penile lesion, reported by Myers *et al*. [3], Caputo *et al*. [4], Yokota *et al*. [5], Meehan *et al*. [6], Seseke F *et al*. [7], and Maekawa *et al*.[8], respectively. Each case was treated by surgical excision, chemotherapy, or steroid ointment, and there are no reports in the literature of recurrence on the penis. Although treatment of vulvar lesions with radiation and surgical excision did not prevent recurrence [9], penile lesions appear to be too sensitive for this type of treatment. In a prior report, distinct Langerhans cell preponderance is shown in the transformation zone of the cervix and the vulva. Langerhans cells are modulated actively in response to certain chemical stimuli, human papillomavirus infection, and cervical intraepithelial neoplasia [6]. It is not proven that the distribution of Langerhans cells or various stimuli to the penis may be related to the rare nature of penile lesions or the good response to the treatment. In the adult cases, there are sole cutaneous involvements of the penis. These cutaneous lesions in adult life may simply represent a limited form of LCH with an excellent prognosis. It is also possible that such lesions are the initial presentation of one of the multi-system instances of LCH [6].

Patients with localized LCH may have a good chance of spontaneous remission and a favorable outcome over a period of months to years [10]. The important factors for predicting recurrent disease and poor prognosis include LCH involving both bone and mucocutaneous tissue, LCH involving both osseous and extraosseous tissues, relapse after treatment of patients with osseous LCH and multi-systemic LCH, >3 bones involvement, the presence of mucous membrane LCH, the presence of hepatosplenomegaly in patients <3 years of age at presentation, pituitary-thalamic axis LCH in the presence of multi-systemic LCH, young age (<5 years) at presentation, and LCH involving 3 or more body systems [2]. This patient had presented LCH at 3 years old and had already relapsed after the treatment for 3 lesions when the penile lesion appeared. Because this may indicate poor prognosis, we will continue to follow the patient closely, to watch for the development of any additional lesions.

## Competing interests

The author(s) declare that they have no competing interests.

## Pre-publication history

The pre-publication history for this paper can be accessed here:
